# Antimony Resistant *Leishmania donovani* but Not Sensitive Ones Drives Greater Frequency of Potent T-Regulatory Cells upon Interaction with Human PBMCs: Role of IL-10 and TGF-β in Early Immune Response

**DOI:** 10.1371/journal.pntd.0002995

**Published:** 2014-07-17

**Authors:** Rajan Guha, Shantanabha Das, June Ghosh, Shyam Sundar, Jean Claude Dujardin, Syamal Roy

**Affiliations:** 1 Division of Infectious diseases and Immunology, CSIR-Indian Institute of Chemical Biology, Kolkata, India; 2 Institute of Medical Sciences, Banaras Hindu University, Banaras, India; 3 Department of Biological Sciences, Antwerp University, Antwerp, Belgium; Institut Pasteur, France

## Abstract

In India the sand fly, *Phlebotomus argentipes*, transmitted parasitic disease termed kala-azar is caused by *Leishmania donovani* (LD) in humans. These immune-evading parasites have increasingly developed resistance to the drug sodium antimony gluconate in endemic regions.

Lack of early diagnosis methods for the disease limits the information available regarding the early interactions of this parasite with either human tissues or cell lineages. We reasoned that peripheral blood mononuclear cells (PBMCs) from healthy human beings could help compare some of their immune signatures once they were exposed for up to 8 days, to either pentavalent antimony sensitive (Sb^S^-LD) or resistant (Sb^R^-LD) *Leishmania donovani* isolates.

At day 2, PBMC cultures exposed to Sb^S^-LD and Sb^R^-LD stationary phase promastigotes had four and seven fold higher frequency of IL-10 secreting monocyte-macrophage respectively, compared to cultures unexposed to parasites. Contrasting with the CD4^+^CD25^−^CD127^−^ type-1 T-regulatory (Tr1) cell population that displayed similar features whatever the culture conditions, there was a pronounced increase in the IL-10 producing CD4^+^CD25^+^CD127^low/−^ inducible T-regulatory cells (iTregs) in the PBMC cultures sampled at day 8 post addition of Sb^R^-LD.

Sorted iTregs from different cultures on day 8 were added to anti-CD3/CD28 induced naïve PBMCs to assess their suppressive ability. We observed that iTregs from Sb^R^-LD exposed PBMCs had more pronounced suppressive ability compared to Sb^S^-LD counterpart on a per cell basis and is dependent on both IL-10 and TGF-β, whereas IL-10 being the major factor contributing to the suppressive ability of iTregs sorted from PBMC cultures exposed to Sb^S^–LD. Of note, iTreg population frequency value remained at the basal level after addition of genetically modified Sb^R^-LD lacking unique terminal sugar in surface glycan.

Even with limitations of this artificial *in vitro* model of *L. donovani*-human PBMC interactions, the present findings suggest that Sb^R^-LD have higher immunomodulatory capacity which may favour aggressive pathology.

## Author Summary

The disease Kala-azar is caused by *Leishmania donovan*i (LD). The disease is characterized by the depression of cellular immune response. In the Indian subcontinent LD parasites are mostly resistant to commonly used antileishmanial drug, like sodium antimony gluconate (SAG). It is known that infection with pentavalent antimony (Sb)-resistant parasites induces aggressive pathology- the cause is still not known. Sb-resistant parasites endowed with unique glycan which may also play an important role in the pathogenesis as following removal of terminal sugar of glycan these parasites behave like sensitive parasites. The diagnosis of the disease is possible after the disease sets in and therefore limited information is available on the host-parasite interaction at the onset of disease. As a surrogate of *in vivo* scenario we studied the interaction between normal human PBMC with Sb-sensitive and Sb-resistant parasites. The Sb-resistant parasites upon interaction with human peripheral blood mononuclear cells (PBMC) *in vitro* produced two distinct inhibitory cytokines, IL-10 and TGF-β. Similar experiment with Sb-sensitive LD induced much less amount of above cytokines. Thus aggressive pathology induced by Sb-resistant LD, may be, in part attributed to production of dual inhibitory cytokines where surface glycan of the parasite may play a decisive role.

## Introduction

Visceral leishmaniasis (VL) or Kala-azar has emerged as a major public health issue in India and neighbouring countries in the last few decades. Pentavalent antimonial compound is the first line drug for therapy of leishmaniasis, with Amphotericin B, Miltefosine and Paramomycin serving as the second line of drugs. Emergence of drug resistance against these drugs has made the situation more alarming for the effective treatment of the disease [1]–[3]. In VL patients, a strong Th1 response is required to prevent the parasitic dissemination while Th2 like cytokines, have shown to aggravate VL [4]–[6]. Suppression of T cell mediated immunity in VL is reported to be mediated by diverse mechanism(s) including i) elicitation of Th2 skewed host immune response [6], ii) effect in macrophage function [7], [8] and iii) regulatory T-cell (Treg) mediated suppression of effector T cell function [9]. However, the detailed mechanism of T cell suppression among VL patients still remains inconclusively elucidated and requires better delineation.

The simplified view that Th1 response leads to cure and Th2 response indicates disease susceptibility cannot fully explain the immune response during active VL. Numerous cytokines from many different cellular sources are involved following *Leishmania* infection and their fine balance may define final outcome of the disease [10]. Remarkable heterogeneity is known to exist among the T cells in terms of their distinct phenotype, function and their proportional participation is believed to dictate the overall T-cell function against parasitic invasion [10], [11]. Suppressive influence of regulatory T cells on effector T cell function suggests their critical involvement in experimental Leishmaniasis [12] and human VL [13]. Subtypes of Treg cells include thymus derived natural Treg cells (nTreg) or adaptive/induced Treg (iTreg). Peripherally induced T regulatory cells (iTreg) may be CD25^+^FoxP3^+^CD127^low/−^ iTregs or other FoxP3^−^ induced T regulatory cells such as Tr1 and TH3 cells [14], [15].Throughout this article we would mention CD4^+^CD25^+^CD127^low/−^ cells as iTreg cells and CD4^+^CD25^−^CD127^−^ cells as Tr1 cells.

Till date all the studies in human VL deals with either active patients or recovered cases of VL. Understanding of immune response on early interaction with the parasite and subsequently the disease onset in host still remains inconclusive. With the emergence of drug resistance, it is imperative to get more conclusive picture of host response during early onset of the disease. This will help us to adopt a better therapeutic approach in controlling human VL, especially for the drug unresponsive cases.

The role of IL-10 as an immunosuppressive molecule is increasingly becoming important in human VL. According to reports, there is an increase in the production of IL-10 among drug-unresponsive cases [6], [16]. *In vitro* infection of murine macrophages and dendritic cells with Pentavalent antimonial resistant (Sb^R^) LD isolates also triggers greater production of IL-10, compared to that of Pentavalent antimonial sensitive (Sb^S^) LD infection [16], [17]. There can be multiple cellular sources of IL-10 in VL patients [18], [19]. However, identities of the distinct cell types contributing to higher IL-10 in drug resistant LD infection remain elusive.

The present study highlights sequential events during the interaction of host PBMCs with Sb^S^ and Sb^R^ LD isolates. Most animal studies with needle injection challenge use very high infection dose (10^6^ to 10^8^ parasites) but in natural transmission the actual dose of parasite is much lower [20]. Using naive human PBMCs in an *in vitr*o set up, we investigated the possible sources of IL-10 during host-parasite interaction. Here we showed that Sb^R^ and Sb^S^-LD isolates sensitise the host cell differentially even at low inoculum. As an immediate interaction response, at early time points several immune cells including monocyte/macrophage, CD4^+^CD25^−^CD127^−^ Tr1 and CD4^+^CD25^+^CD127^low/−^ iTreg cells produce IL-10 in both Sb^S^ and Sb^R^-LD infection. Interestingly, at a later time point, CD4^+^CD25^+^CD127^low/−^ iTreg cells contribute towards the enhanced suppression of effector cells in Sb^R^-LD infection by generating more IL-10 and TGF-β. This study analysed the differential immune modulation by Sb^R^-LD isolates during their interaction with human PBMCs.

## Materials and Methods

### Ethics statement

Use of Human subjects was approved by “*Ethical Committee of Human Subjects*” of Indian Institute of Chemical Biology. Blood were drawn from normal healthy individuals after their written informed consent.

### Parasites

Stationary phase promastigotes of well characterized Pentavalent antimonial sensitive (Sb^S^) *Leishmania donovani* strain AG83 (MHOM/IN/83/AG83), 777 (MHOM/IN/09/BHU777/0), 816 (MHOM/IN/10/BHU816/1) and Pentavalent antimonial resistant (Sb^R^) *Leishmania donovani* strain 138 (MHOM/IN/2005/BHU138), 575 (MHOM/IN/09/BHU575/0), 814 (MHOM/IN/10/BHU814/1) were used [16].We selected the drug resistant parasites depending on different parameters like their IC50 values, surface expression of multidrug receptors, different biophysical properties of the parasite membranes; the details of which is described elsewhere [16]. Parasites were harvested on day 5 of culture for *in vitro* evaluation of the response of peripheral blood mononuclear cells (PBMCs).GALT Knock down Sb^R^-*Leishmania donovani* (KDSb^R^-LD) parasites were generated as described previously [21].

### Isolation and *in vitro* culture of mononuclear cells

Fifty millilitres of human blood was collected during each blood draw from healthy individuals after their informed consent. The donors reported to have never suffered from leishmaniasis and have never travelled to leishmaniasis endemic regions. They were all tested rK39 negative and considered as *Leishmania* naïve. The PBMC were isolated from heparinized venous blood by passage over a Ficoll-Hypaque 1.077 (Sigma-Aldrich) gradient. PBMC were washed three times and resuspended at a concentration of 2.5×10^6^ cells/mL in complete medium consisting of RPMI 1640 medium supplemented with 2 mM L-glutamine, penicillin (100 U/mL), gentamicin (100 µg/mL), and 10% heat-inactivated human AB serum (Sigma-Aldrich). The cells were plated in 24-well tissue culture plates (Costar, Corning, NY) in a volume of 1 mL/well. *Leishmania donovani* promastigotes were opsonised for 1 h at 37°C in RPMI 1640 containing 10% heat-inactivated AB^+^ serum in a humidified 5% CO_2_ atmosphere, washed, and resuspended in complete RPMI 1640. Opsonisation was conducted to optimize infection and approximate the vivo conditions. PBMC's were cultured with 2.5×10^5^/mL of opsonised promastigotes (approximate parasite to monocyte ratio, 1∶1) or medium alone. Supernatants were harvested at 1, 2, 3 and 8 days post parasite addition for cytokine quantification.

For parasite burden enumeration the same assay was set up on coverglass. On early (day 2) and later time point (day 8), coverglasses were washed, fixed with methanol followed by staining with Giemsa. Stained coverglasses were then mounted on a slide and observed under light microscope.

### Cytokine detection

Cytokines were quantified in supernatant samples obtained from cultures at 24, 48, 72 hrs and day 8 using the BD human Th1/Th2 cytokine kit II (BD Pharmingen) and BD CBA human inflammatory kit. To double check the results, IFN-γ and IL-10 were also measured by enzyme-linked immunosorbent assay (ELISA) [22]. IL-27 and TGF-β levels were determined by using human DUO set (R and D system). CBA analysis was done using FCAP array in a BD FACS ARIAII flow cytometer.

### FACS analysis

Cells were stained with relevant antibodies on ice for 30 minutes in PBS buffer containing 2% FCS and 0.1% sodium azide. Before staining for surface markers, cells were incubated with Fc Blocker (CD16/CD32) for 30 minutes to minimize non-specific staining. Cells were washed twice before being analysed by BD FACS ARIA II flow cytometer. Live cells were gated based on forward and side scatter profiles and based on exclusion with live/dead aqua marker (Invitrogen). The following antibodies were used for staining: anti-human CD3 PerCP, CD4 APC or CD4 PE-Texas red, CD25 APC-Cy7, CD127 PE-Cy7, GARP PE (All from BD Biosciences) and anti-human Latency associated peptide (LAP)-TGF-β1 Alexa Fluor 488 (R and D systems). Recombinant human LAP (rLAP) that associates with TGF-β1 was purchased from R and D systems. Analysis was performed using FlowJo software (Tree Star).

The Tr1 and iTreg cells were sorted on the BD FACS ARIAII Flow Cytometer aseptically for further experiments. 2.5 million PBMCs from each well of tissue culture plate were sorted in approximately 5 minutes. Sorted cells were collected in 12×75 mm polypropylene tubes pre-coated with human AB serum and containing complete RPMI-1640 with 10% human AB serum.

### IL-10 secretion assay

IL-10 secretion assay was used for evaluation of IL-10 production from different subset of cells (Milteny Biotech) according to manufacturer's protocol. Briefly PBMCs were harvested after *in vitro* infection with different LD parasites at early (48 hr p.i) and late time point (8 day p.i) cells These PBMC's were then labelled with IL-10 catch reagents and kept for 45 minutes to 1 hr in rocking condition at 37°C.During this time period secreted IL-10 binds to the IL-10 catch reagent. After that the cells were stained with PE labelled anti-IL-10 detection antibody along with fluorescent labelled anti-human CD4, anti-human CD3, anti-human CD14, anti-human CD127, anti-human CD25 antibody and acquired on FACS ARIA II flow cytometer. Data analysis was performed using FlowJo software (Tree Star).

### Quantitative PCR

Total RNA from the sorted cells was isolated and cDNA was synthesized using SuperScript VILO cDNA Synthesis Kit (Invitrogen). 50–100 ng of total RNA was used for the synthesis of cDNA. RT^2^ qPCR Primer Assay Primers were purchased from SA Biosciences. Real-time quantitative PCR was conducted as per the protocol described earlier [23], [24]. Briefly, it was carried out with 12.5 µl of SYBR green PCR master mix (ABI), 1 µl of cDNA from RT reaction mix, and gene specific primers in a final volume of 25 µl. PCR was conducted under the following conditions: initial denaturation at 95°C for 10 min followed by 40 cycles, each consisting of denaturation at 95°C for 15 s, annealing at 60°C for 1 min, and extension at 72°C for 40 s per cycle using the ABI 7500 Real time PCR system. cDNAs from normal uninfected controls were used as “comparator samples” for quantification of those corresponding to test samples. All quantifications were normalized to the housekeeping gene 18SrRNA. A no-template control c-DNA was included to eliminate contaminations or nonspecific reactions. The cycle threshold value was defined as the number of PCR cycles required for the fluorescence signal to exceed the detection threshold value background noise [25]. Differences in gene expression were calculated by the comparative CT method [24]. This method compares test samples to a comparator sample and uses results obtained with a uniformly expressed control gene (18SrRNA) to correct for differences in the amounts of RNA present in the two samples being compared to generate a ΔCT value. Results are expressed as the degrees of difference between ΔCT values of test and comparator samples.

### Co-cultures and proliferation assays

To verify the regulatory function of iTreg cells (CD4^+^CD25^+^CD127^low/−^), iTreg cells were isolated by sorting from 8 days Sb^R^, Sb^S^-LD infected and unstimulated PBMC's. This isolated Treg cells were co-cultured with autologous freshly isolated PBMC at different iTreg: responder ratio in 96-well U-bottom plates in the presence of anti-CD3/CD28/CD2 mixed beads (T Cell Activation/Expansion Kit, Milteny Biotech) at 37°C and 5% CO2. On day three, 4 h before the end of incubation period, BrdU solution was added to the cells. BrdU will be incorporated in the proliferating cells. The level of BrdU incorporation was measured according to manufacturer's protocol (Millipore) and the absorbance was measured at 450 nm using ELISA plate reader (DTX 800 multimode detector: Beckman Coulter).The suppression index was calculated as described earlier [26], when the assay was performed in the presence of neutralizing IL-10 (25 µg/ml) or rLAP (20 µg/ml), the reagents were added at the start of the assay in the co-culture experiment.

### Statistical analysis

All experiments were done on blood from individual donor and a representative/pooled data are presented, interassay variation being within 5–10%. All graphs and statistical analyses were generated in GraphPad Prism 5.01 (GraphPad, San Diego,CA). Student's two tailed paired *t* test, Mann-Whitney test and Wilcoxon matched-pairs test was used to determine differences between different groups with 95% confidence intervals. P values less than 0.05 were considered to be significant for all analyses.

## Results

### Sb^S^-LD or Sb^R^-LD induced IL-10 production from normal PBMC

**Figure 1:**
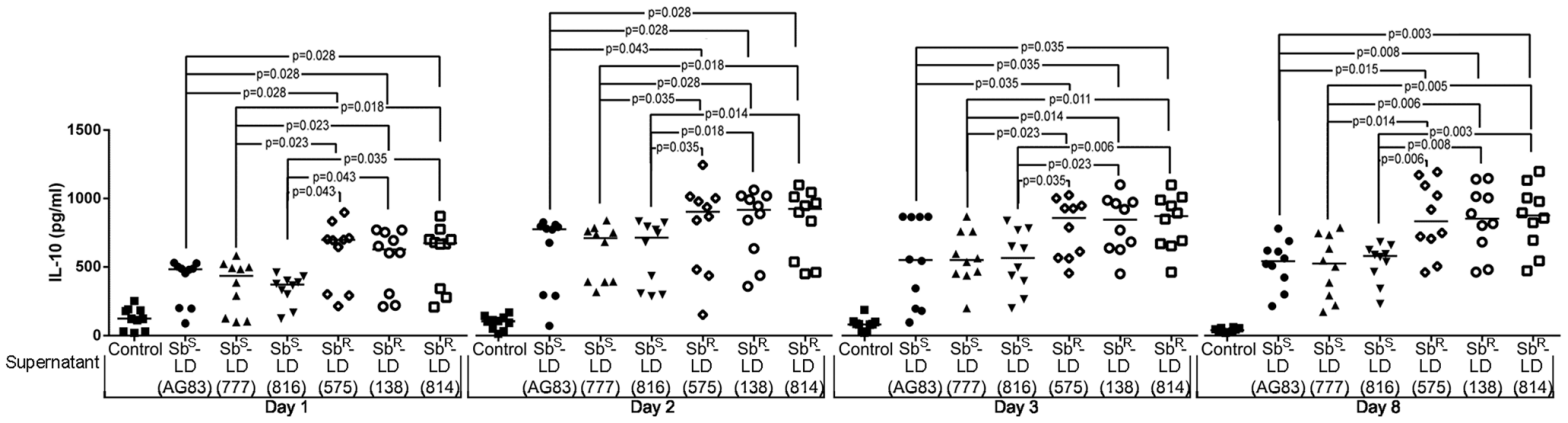
Sb^R^-LD parasites induce greater IL-10 production than Sb^S^-LD isolates. Kinetics of IL-10 production during early interaction with different Sb^S^ and Sb^R^-LD isolates. Briefly, freshly isolated PBMCs were incubated with Sb^S^ and Sb^R^-LD isolates and culture supernatants (Sb^S^ and Sb^R^-Sup) were isolated on day- one, two, three and eight, and IL-10 level was measured by ELISA/CBA. The level of IL-10 production at different time points are represented (day one, two, three and eight), n = 10, median values of different time points were indicated. Data were analysed by the Mann-Whitney test, and levels of significance are indicated by P values.

The kinetics of IL-10 production in culture supernatants from PBMC upon co-culturing with Sb^S^-LD or Sb^R^-LD was quantified. The experiment was conducted up to day 8 beyond which the culture could not be maintained **(** **Figure 1** **)**. The results were plotted as individual value and expressed as median. There is statistically significant enhancement in IL-10 production from PBMC in response to in vitro parasite challenged with respect to unstimulated control **(** **Figure 1** **)**.The maximum IL-10 production was noted on day 2 **(** **Figure 1** **,** **Table 1** **)**. There was essentially 50% increase in IL-10 production in Sb^R^-LD driven PBMC culture supernatants (Sb^R^-supernatant) as compared to Sb^S^-LD driven PBMC culture supernatant (Sb^S^-supernatant) from day 1–3. However, on day 8, Sb^S^-supernatant showed more substantial decrease in IL-10 production as compared to resistant counterpart **(** **Table 1** **)**. In this investigation three Sb^R^-LD (LD-575, LD-138 and LD-814) and three Sb^S^-LD (AG83, LD-777 and LD-816) strains were used. Since, Sb^R^-LD-575, Sb^R^-LD-138 and Sb^R^-LD-814 induced essentially similar level of IL-10 production as evident from the median values **(** **Figure 1** **,** **Table 1** **)**, we used Sb^R^-LD-575 (henceforth defined as Sb^R^-LD) as the representative of pentavalent antimony (Sb)-resistant parasites for further studies. Similarly AG83 (henceforth defined as Sb^S^-LD) was used as the representative of pentavalent antimony (Sb)-sensitive parasites. We focused our studies on day 2 (early time point) and day 8 (late time point) for the rest of the investigation.

**Table 1:** Median values of IL-10 production (pg/ml) at different time points.

	CONTROL	Sb^S^-sup. (AG83)	Sb^S^-sup. (BHU-777)	Sb^S^-sup. (BHU-816)	Sb^R^-sup. (BHU-575)	Sb^R^-sup. (BHU-138)	Sb^R^-sup. (BHU-814)
**DAY 1**	121.8	483.4	434.2	371.0	696.8	627.5	673.7
**DAY 2**	105.3	783.5	716.7	721.0	911.4	925.4	932.5
**DAY 3**	82.48	553.9	554.5	569.5	865.4	852.6	879.3
**DAY 8**	40.20	547.5	529.7	585.6	842.0	861.5	885.0

The median values of IL-10 production at different time points are tabulated. Freshly isolated PBMCs were incubated with different Sb^S^ and Sb^R^-LD isolates and culture supernatants (Sb^S^ and Sb^R^-Sup) were isolated on day- one, two, three and eight, and IL-10 level (pg/ml) was measured by ELISA/CBA.

### CD14^+^ cells as a source of IL-10 in response to Sb^S^-LD and Sb^R^-LD

**Figure 2:**
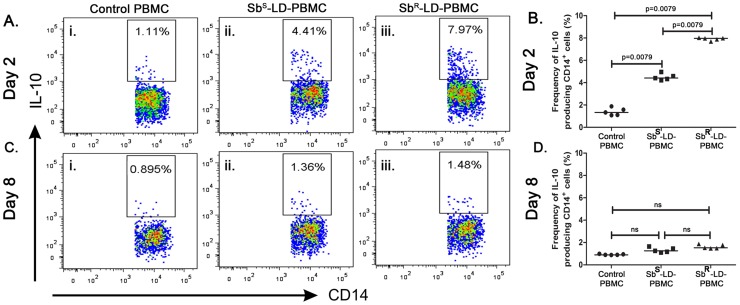
CD14^+^ monocyte/macrophage cells produce IL-10 during initial interaction. Production of IL-10 from CD14^+^ monocyte/macrophage cells. The frequencies of CD14^+^IL-10^+^ cells from Sb^S^-LD-PBMC and Sb^R^-LD-PBMC at different time points are represented. **A–B**. Percentage of IL-10 producing CD14^+^ cells on day two and **C**–**D**; on day eight. Pseudo colour plot indicate one representative data set. Data were analysed by the Mann-Whitney test, and levels of significance are indicated by P values.

The frequencies of CD14^+^IL-10^+^ cells were enumerated in PBMCs in response to Sb^S^-LD or Sb^R^-LD challenge. It was observed that in response to Sb^S^-LD and Sb^R^-LD challenge, frequency of CD14^+^IL-10^+^ cells increased four and seven folds on day2 respectively compared to control **(** **Figure 2A, B** **)**. The frequency of CD14^+^IL-10^+^ was decreased on day 8 as compared to day 2, regardless of input parasites **(** **Figure 2C, D** **)**. Interestingly, one could see IL-10 in the culture supernatants on day 8 suggesting there are other potential sources of IL-10.

### Induction of CD4^+^CD25^−^CD127^−^ (Tr1) and CD4^+^CD25^+^CD127^low/−^ (iTreg) cells in response to Sb^S^-LD and Sb^R^-LD

**Figure 3:**
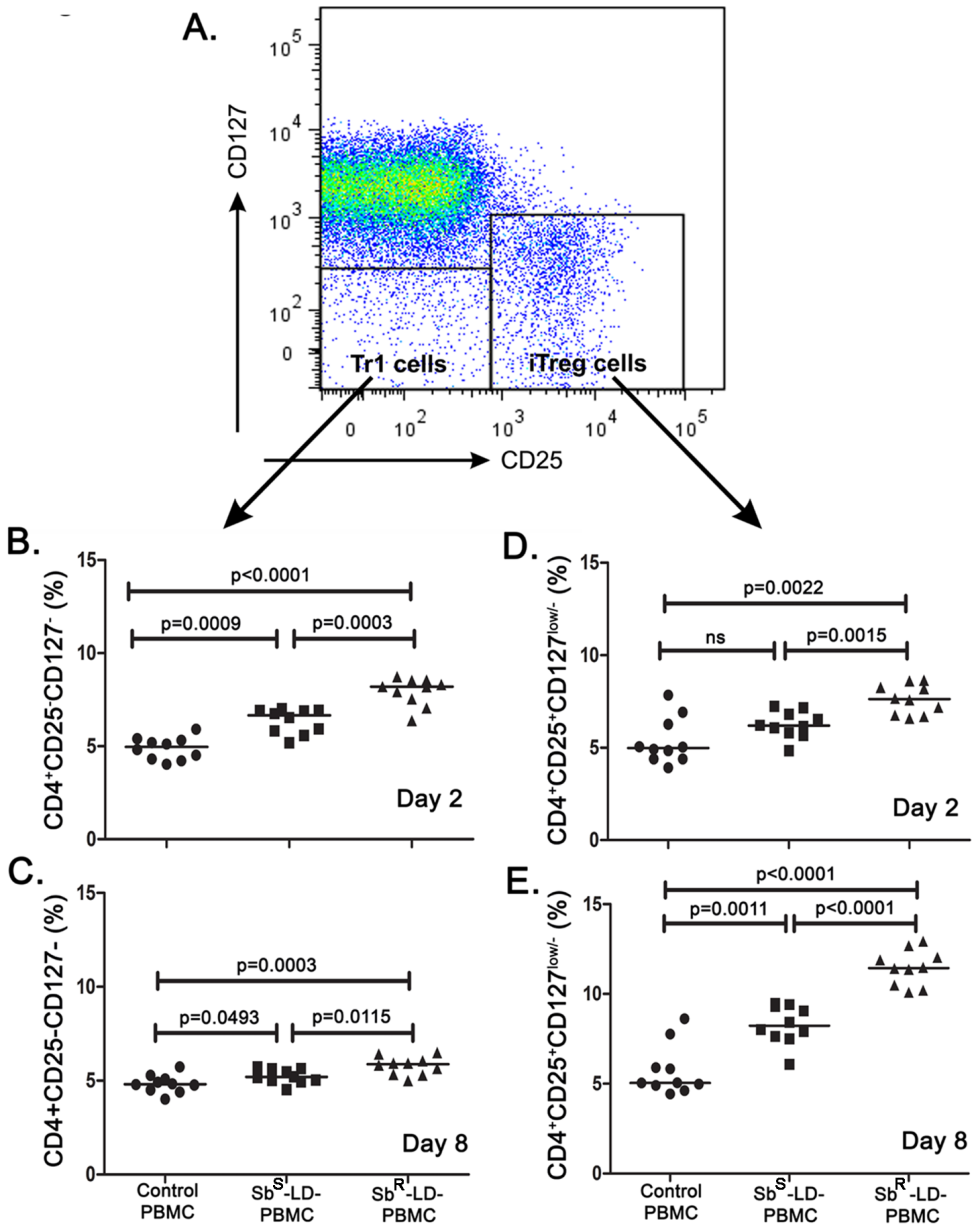
Different regulatory T-cells are induced upon interaction with *Leishmania donovani*. Percentage of Tr1 (CD4^+^CD25**^−^**CD127^−^) and iTreg (CD4^+^CD25**^+^**CD127^low/−^) cells among gated CD4^+^ cells (n = 10). Briefly, freshly isolated PBMCs were incubated with Sb^S^ and Sb^R^-LD isolates and after day 2 and day 8 cells were stained and percentage of Tr1 and iTreg cells were analysed by Flow Cytometry. Lymphocytes were gated based on forward and side scatter profiles, and live cells were identified on the basis of exclusion of LIVE/DEAD Aqua dye (Invitrogen), followed by CD3^+^ and subsequently on CD4^+^ cells. **A**. CD4^+^ cells were further gated based on the expression of CD25 and CD127 to identify Tr1 (CD4^+^CD25**^−^**CD127^−^) and iTreg (CD4^+^CD25**^+^**CD127^low/−^) populations. **B–C**. Percentage of Tr1 cells on day two and eight respectively, **D–E**. Percentage of iTreg cells on day two and eight respectively. Data were analysed by the Mann-Whitney test, and levels of significance are indicated by P values.

Based on earlier reports [27], here we have used IL-7 receptor (CD127) to define the T regulatory cells. CD4^+^CD25^+^CD127^low/−^ cells were designated as iTreg cells and CD4^+^CD25^−^CD127^−^ cells were designated as the Tr1 cells (**Figure 3A**).

On day 2, a significant increase in Tr1 cells was noted in PBMC culture in response to Sb^S^-LD (Sb^S^-LD-PBMC) or Sb^R^-LD (Sb^R^-LD-PBMC). In the latter case the response was significantly higher as compared to former **(** **Figure 3B** **)**. However, there was low but significant increase in Tr1 cells was noted regardless of input parasites on day 8 **(** **Figure 3C** **)**. The frequencies of iTreg cells on day 2, in Sb^S^-LD-PBMC or in Sb^R^-LD-PBMC, were essentially similar as observed in the case of Tr1 cells **(** **Figure 3D** **)**. However, there was a significant increase in iTreg cells in Sb^S^-LD-PBMC and Sb^R^-LD-PBMC on day 8 as compared to normal but the Sb^R^-LD triggered significantly higher frequency of iTregs than the Sb^S^-LD **(** **Figure 3E** **)**.

### Frequencies of IL-10 producing Tr1 and iTreg cells

**Figure 4:**
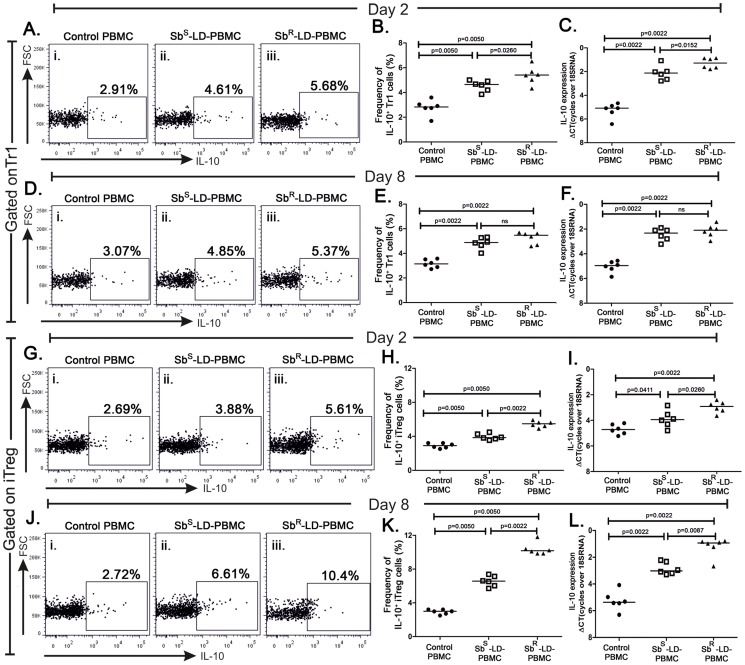
IL-10 producing Tr1 and iTReg cells are induced in response to Sb^S^ and Sb^R^-LD. Percentages of IL-10 producing Tr1 and iTreg cells from Sb^S^-LD-PBMC and Sb^R^-LD-PBMC at early and late time point are represented. **A, B**. Percentage of IL10^+^Tr1 cells on day two, **C**. Real-Time RTPCR of IL-10 mRNA level in sorted population of Tr1 cells on day two. **D, E**. Percentage of IL10^+^Tr1 cells on day eight, **F**. Real-Time RTPCR of IL-10 mRNA level in sorted population of Tr1 cells on day eight. **G, H**. Percentage of IL10^+^iTreg cells on day two, **I**. Real-Time RTPCR of IL-10 mRNA level in sorted population of iTeg cells on day two. **J, K**. Percentage of IL10^+^iTreg cells on day eight, **L**. Real-Time RTPCR of IL-10 mRNA level in sorted population of iTreg cells on day eight. Dot plots indicate one representative data set. Data were analyzed by the Mann-Whitney test, and levels of significance are indicated by P values.

The frequencies of IL-10 producing Tr1 cells (IL10^+^ Tr1) and iTreg (IL-10^+^ iTreg) in Sb^R^-LD-PBMC and Sb^S^-LD-PBMC were enumerated. In general, the frequencies of such cells in both the compartments increased significantly regardless of input parasites as compared to their respective controls **(** **Figure 4A–L** **)**. There is low but significant difference in IL-10^+^ Tr1 cells between Sb^S^-LD-PBMC and Sb^R^-LD-PBMC on day 2 (**Figure 4A–C**). But on day 8, difference in IL10^+^ Tr1 cells between Sb^S^-LD-PBMC and Sb^R^-LD-PBMC cannot be seen **(** **Figure 4D–F** **)**. On the other hand, there was a significant difference in IL-10^+^ iTreg between Sb^S^-LD-PBMC and Sb^R^-LD-PBMC which was higher in later case in both the time points **(** **Figure 4G–L** **)**. Most interestingly, there is a remarkable difference in IL-10^+^ iTreg between Sb^S^-LD-PBMC and Sb^R^-LD-PBMC where frequency was much higher in the latter case **(** **Figure 4J–L** **)** on day 8. The results observed by flow cytometry analysis were corroborated well with the quantitative PCR data for IL-10 signal **(** **Figure 4C, F, I, L** **)**.

### iTreg cells from Sb^R^LD driven PBMC show profound suppressive ability

**Figure 5:**
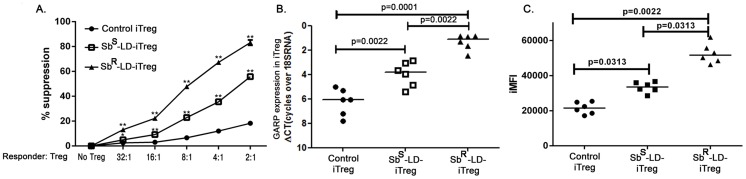
Isolated iTregs are active and shows pronounced suppressive ability. **A**. Percentage suppression analysed after co-culturing isolated iTreg cells (described earlier) at day eight with autologous freshly isolated PBMC at different Treg: responder ratio. Expression of GARP/LRRC32 in iTreg cells. **B**. mRNA expression level of LRRC32 gene in sorted iTreg cells with median values indicated and **C**. iMFI values was calculated by multiplying the frequency of GARP protein expressing iTregs and their mean fluorescence intensity. Data were analyzed by the Mann-Whitney test, and levels of significance are indicated by P values. Groups Sb^S^-iTreg and Sb^R^-iTreg were compared to control iTregs. “*” indicates 0.0001<P<0.05 and “**” indicates P<0.0001.

To assess their suppressive ability, if any, sorted iTreg cells derived from day 8 culture were co-cultured with freshly isolated autologous PBMCs as responders, in the presence of anti-CD3 and anti-CD28 beads. It was observed that T-cell proliferation decreased as a function of iTreg number and maximum suppression was achieved at a ratio of 2∶1 (responder : iTreg) **(** **Figure 5A** **)**. The iTregs isolated from Sb^R^-LD-PBMC showed about 80% suppression whereas similar iTregs from Sb^S^-LD-PBMC showed about 55% suppression. In contrast, iTregs derived from normal subjects only minimally suppressed the proliferation of responder cells **(** **Figure 5A** **)**.

In human, expression of a transmembrane protein, glycoprotein A repetitions predominant (GARP or LRRC32) selectively identifies activated T-regulatory cells [28]. We measured the surface expression of GARP in iTreg cells in Sb^R^-LD-PBMC and Sb^S^-LD-PBMC on day 8. When GARP expression was monitored in the iTregs at the mRNA level, substantial difference was detected between Sb^S^-LD-PBMC and Sb^R^-LD-PBMC **(** **Figure 5B** **)**. Frequency of GARP expressing iTreg cells and the level of its expression was taken into account when calculating iMFI (integrated MFI) values. It displays that iMFI values for GARP expressing iTreg cells are noticeably higher in Sb^R^-LD-PBMC compared to Sb^S^-LD-PBMC samples **(** **Figure 5C** **)**.

### Induced T regulatory cells (iTreg) mediates suppression in a IL-10 and TGF-β dependent manner in Sb-R infection

**Figure 6:**
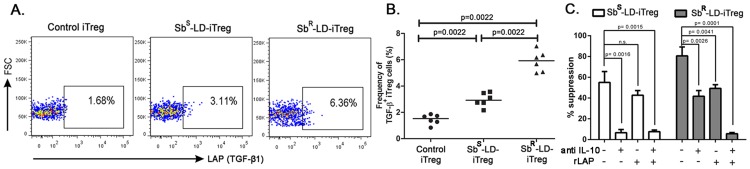
iTReg from Sb^R^-LD-PBMC mediates their suppressive activity through IL-10 and TGF- β. Percentages of rLAP (TGF-β1) containing iTreg cells from Sb^S^-LD-PBMC and Sb^R^-LD-PBMC at day eight are represented. **A, B**. Percentage of LAP (TGF-β1)^+^ iTreg cells on day eight. Pseudo colour plot indicate one representative data set. **C**. Suppression assay in presence of neutralizing IL-10 antibody and/or rLAP. Data were analysed by Mann-Whitney test and two-tailed paired student t test, and levels of significance are indicated by P values.

We investigated expression of cell surface LAP/TGF-β1 in LD infection, as GARP in activated Treg is known to remain associated with it. We were able to detect membrane bound LAP/TGF-β1 in iTreg cells. The percentage of LAP/TGF-β1^+^ iTreg cells being significantly higher in Sb^R^-LD-PBMC (Median 5.925%) in contrast to Sb^S^-LD-PBMC (Median 2.925%) **(** **Figure 6A, B** **)**.

Based on the fact that iTreg of Sb^R^-LD-PBMC expressed both IL-10 and TGF-β, we endeavoured to study the effects of neutralizing/blocking antibodies to above mentioned cytokines on the iTreg mediated suppression of T-cell repertoire at day 8. It was observed that the presence of anti-IL-10 antibody alone almost abolishes the suppressive function of iTreg from Sb^S^-LD-PBMC **(** **Figure 6C** **)**. Exactly identical experiment was performed with iTreg from Sb^R^-LD-PBMC. Here, addition of anti-IL-10 induced about 50% inhibitions whereas; recombinant human LAP (rLAP), which neutralizes active TGF-β1, induced about 45% inhibition of iTreg's suppressive capacity. Addition of combination of anti-IL-10 and rLAP led to complete inhibition of these iTreg's suppressive ability **(** **Figure 6C** **)**. It is to note that apart from TGF-β1, the rLAP can also neutralize other active isoforms of TGF-β. Hence involvement of other isoforms of TGF-β cannot be ruled out.

### Role of surface glycans in differential immune modulation

**Figure 7:**
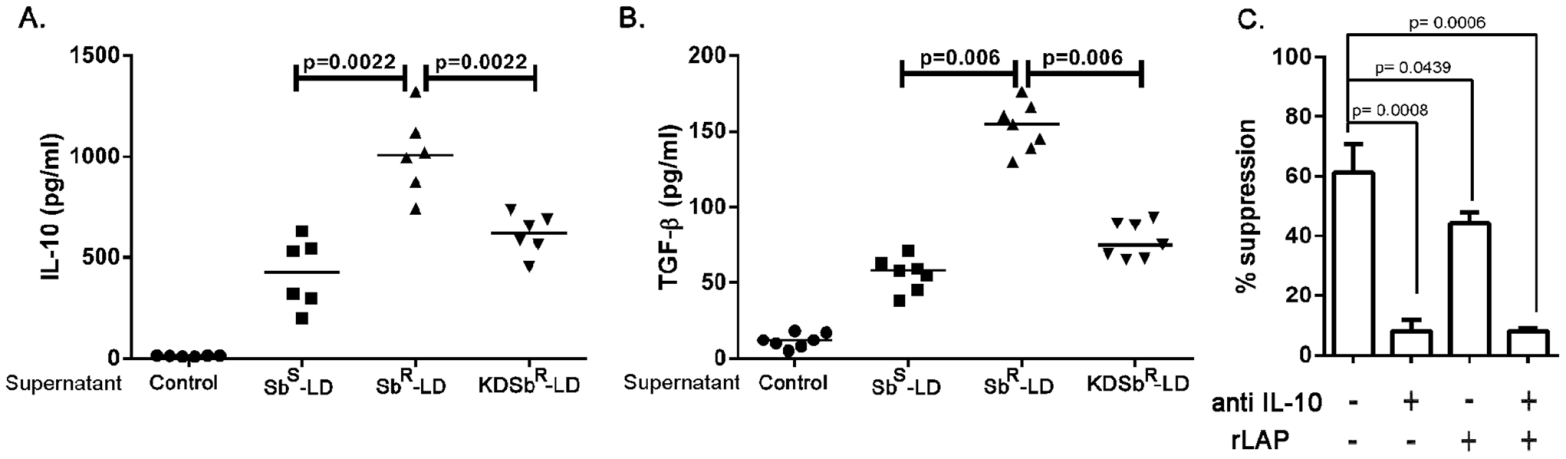
GalT Knock down parasites (KDSb^R^-LD) behaves like Sb^S^-LD isolates. **A**. Production of IL-10, **B**. TGF-β from KDSb^R^, Sb^S^ and Sb^R^-Sup. For TGF-β measurement the culture supernatants were acidified followed by neutralization, the level of TGF-β was measured by ELISA. **C**. Suppression assay performed in presence of neutralizing IL-10 antibody and/or rLAP using iTreg from KDSb^R^-LD-PBMC at day eight. Data were analysed by Mann-Whitney test and two-tailed paired Student t test, and levels of significance are indicated by P values.

Our group earlier showed that expression of a unique glycan with *N-acetylgalactosamine* as a terminal sugar on Sb^R^-LD plays an important part in up-regulation of IL-10 and MDR1 in infected macrophages and removal of this glycan makes this parasite to behave like their antimony sensitive counterpart [21]. Here by using the galactosyl transferase knock down parasite (KD-Sb^R^-LD) we got the level of IL-10 almost close to the amount we observed in infection with Sb^S^-LD **(** **Figure 7A** **)**, which indicates towards a possible role of this glycan in the observed differential immune response. Our earlier experiments showed that rLAP reverses the suppressive effect of iTReg cells from Sb^R^-LD-PBMC. Here; we acid neutralize the culture supernatant and measured the TGF-β from culture supernatants by ELISA at day 8. Our results showed that Sb^R^-LD driven PBMC culture supernatants produced higher level of TGF-β compared to that of Sb^S^-LD driven PBMC culture supernatants but the production of TGF-β from knock down parasite (KD- Sb^R^-LD driven PBMC culture supernatants) was considerably low and was comparable to Sb^S^-supernatant **(** **Figure 7B** **)**. Next we did the same suppression assay performed earlier by isolating iTreg from KD-Sb^R^-LD infection and addition of neutralizing IL-10 alone abolished the suppressive activity compared to that of iTreg from wild type Sb^R^-LD-PBMC **(** **Figure 7C** **)**.

Another important thing to consider is the level of infection induced by these parasites in human PBMC. For that we set up the same experiment on a coverglass. On day 2 and day 8 we measured the level of infection in the adherent cells. We found that there was hardly any difference in the level of infection between Sb^S^-LD, Sb^R^-LD and KD-Sb^R^-LD though the median values indicated a slightly higher level of infection load in the case of Sb^R^-LD infection (**Figure S1**).

## Discussion

There is very little information available about the early immune responses (first few days) when human host is infected with *L. donovani*. We adopted an approach proposed by Rogers *et al* that allows us to study such interactions over a defined period of time in an *in vitro* setting [29], [30]. We did this study with both Sb^S^ and Sb^R^-LD isolates; it allowed us to dissect the differences in their immune modulation capacity during their interaction with the host PBMCs. Infectious dose of the parasite transmitted by the sand flies varies considerably [31]–[33], so choosing an inoculums size was a challenging task. We used opsonised live LD parasite (approximate parasite to monocyte ratio, 1∶1) to get as closely as possible to the *in vivo* scenario in human [29], [34], as reports suggests that the response of PBMC's to dead versus live parasites differs significantly [35]. These studies are not possible with the PBMCs from *Leishmania* infected, cured or exposed individuals, as we are unaware about the time of infection, dose, frequency or sometimes also the species of the parasites [29], [30], [36]. If we use PBMCs from these study subjects, then the immune response will be more close to secondary immune response [29], [30]. It is noteworthy that use of PBMCs from asymptomatic individuals would have been interesting. A high incidence of asymptomatic cases indicates that many individuals are indeed capable of mounting effective immune response to keep *Leishmania* infection in check. Prognosis of individual markers responsible for asymptomatic infection at individual level is still lacking. There are reports that unlike individuals with active disease, PBMCs from some of the asymptomatic individuals indeed respond to antigens of *L. chagasi* with proliferative response and secretion of cytokines such as IL-2, IFN-γ and IL-12 which are implicated in anti-leishmanial response. Also level of disease promoting cytokine IL-10 following *L. chagasi* antigen encounter remains lower in asymptomatic individuals compared to patients with active pathology [37], [38]. Earlier reports showed that following stimulation of whole-blood with *L. donovani* soluble *Leishmania* antigen (SLA), cells from individuals with active disease responds by secretion of both IFN-γ and IL-10 but asymptomatic individuals do not produce the disease promoting IL-10 [39].

Diverse reports exist about the source of IL-10 in *Leishmania* infection. There is a lot of discrepancy about the source of IL-10 in various previous studies with VL patients or using murine model. In a non-healing mouse model of Leishmaniasis, high frequency of IL-10 producing regulatory T cells (CD4^+^CD25^+^FoxP3^+^) is observed at local site [9]. Recent study by Nylen *et al*, 2007 has evidently shown the IL-10 mediated suppression of immunity among patients suffering from VL [19]. However, in a subsequent study the source of IL-10 among VL patient was identified as CD25^−^FoxP3^−^ cells, not the CD25^+^FoxP3^+^ Treg cells as presumed on the basis of data obtained from some studies [18]. Regulatory T-cells are part of body's physiologic regulatory mechanisms used by the immune system to maintain homeostasis for preventing autoimmunity and temper inflammation after infection or injury [15].T-regulatory cells are considered to be pivotal mediators of peripheral tolerance and immune suppression [15]. Our studies revealed the role played by different T-regulatory cells during early interactions of host with Sb^R^-LD and Sb^S^-LD parasites. CD4+CD25^−^CD127^−^ Tr1 cells produce IL-10 and are constant source of IL-10 at both early and late time point. Increase in the level of IL-27 in interactions with both Sb^S^-LD and Sb^R^-LD isolates correlates with the generation of IL-10 producing Tr1 cells (**Figure S2**). But interestingly the percentage of CD4^+^CD25^+^CD127^low/−^ iTreg cells increases at late time point in Sb^R^-LD infection and these cells also contributes to IL-10 production considerably. Sb^S^-LD infections also result in production of IL-10 from iTreg cells but not to same extent. Earlier studies from our group have shown that Sb^R^-LD infection generates more IL-10 from both macrophages and dendritic cells [16], [17]. Verreck *et al.* reported the existence of different macrophage population derived from same CD14^+^ monocytic lineage [40], one such population produces high levels of IL-10 and has a capacity to induce differentiation of T regulatory cells [41]. These reports and our observation indicate a possible role of these anti-inflammatory macrophages in the induction of Treg cell during Sb^R^-LD infection. Walther *et al* reported that there is a strong co-relation between rapid growths of virulent strains of malaria with increase in the frequency of CD4^+^CD25^+^CD127^low/−^ T regulatory cells [42]. Generation of iTreg cells or expansion of nTreg cells with concomitant IL-10 production in Sb^R^-LD infection may be one of reasons behind the persistence of Treg cells even after successful chemotherapy as reported by Rai *et al* [13]. Many chronic infections are associated with this increase in Treg cell numbers [43], [44]. Presence of these Treg cells during these infections becomes an important barrier for any vaccination or other treatment strategies [45]. A successful vaccine against Leishmaniasis is still elusive despite several recent encouraging reports [46]–[48] and success of such vaccine depends on the IL-10 production by Treg cells [49]. Presence of Treg cells and associated IL-10 production is reported in reactivation of disease in the form of PKDL [50].

We have shown here that these iTreg cells are capable of suppressing the proliferation of autologous cells. Enhanced suppressive ability of the iTreg cells isolated from Sb^R^-LD infection emphasizes the role played by these cells in the suppression of effector T cells in drug-unresponsive cases. Reports suggest that activated T regulatory cells express a surface molecule called glycoprotein A repetitions predominant (GARP) [51].Our data confirms the upregulation of this activation marker in the iTreg cells from Sb^R^-LD infection, which further substantiates the suppressive role of these active iTreg cells in Sb^R^-LD infection.

T-regulatory cells exert their suppressive function in many ways, by secreting soluble factors, in contact dependent manner or by quenching growth factors [52]. Sb^R^-LD infection enhances IL-10 production indicating its importance in observed immune suppression. But addition of neutralizing IL-10 can't rescue the suppressive effect of iTreg cells from Sb^R^-LD-PBMC. GARP acts as a receptor for membrane bound TGF-β in activated Tregs [53]. This prompts us to check whether TGF-β also has some role in this immune suppression. Addition of rLAP, which binds to TGF-β1 together with anti-IL-10, does rescue the suppressive effect of iTreg from Sb^R^-LD-PBMC. On the other hand addition of neutralizing IL-10 alone can almost entirely inhibit suppressive effect of iTreg isolated from Sb^S^-LD-PBMC. Higher level of TGF-β from acid neutralized Sb^R^-supernatant and higher expression of surface bound TGF-β in iTreg isolated from Sb^S^-LD-PBMC proves the association of IL-10 and TGF-β in the iTreg mediated immune suppression in Sb^R^-LD infection. Activated Treg cells induce the differentiation of naïve cells to FoxP3^+^ Treg cells in a TGF-β-dependent cell contact-dependent manner [54].The continuous generation of suppressive iTreg cells in Sb^R^-LD infection may be responsible for greater immune-suppression and persistence of the parasite after successful chemotherapy. The terminal sugar residue N-acetylgalactosamine present on the surface of Sb^R^-LD may have some role in their differential immune response as knocking down of galactosyl transferase enzyme, which adds these residues, reverses this differential immune response and Sb^R^-LD behaves like their sensitive counterpart as shown by us in our earlier studies [21] and in this present study. Furthermore previous study with KD-Sb^R^-LD parasites resulted in significantly less parasite burden in BALB/c mice compared to infection induced by Sb^R^-LD [21]. It is a well-known fact that glycans can interact with dendritic cells/macrophages to produce cytokines that can modulate T-cell response [55], [56]. But in our case, actually how the surface glycoprotein affects the antigen presenting cells to induce differential T-cell response needs further careful and thorough investigation. Our earlier report showed that the Sb^R^-LD isolates expressed surface glycoconjugates with a terminal sugar N-acetyl-D-galactosaminyl residue, which was almost absent in Sb^S^-LD [21]. The induction of more potent iTregs secreting both IL-10 and TGF-β as effectors by Sb^R^-LD parasites was abrogated in case of KD-Sb^R^ -LD driven iTregs, as reported in this work. These results further establish that the terminal sugar present in the Sb^R^-LD isolates is one of the key components responsible for differential immune-modulation following interaction with human PBMCs. Earlier reports suggest that Sb^R^-LD infection may lead to greater infection load and more aggressive disease pathology [57]. But this observed differential immune-modulation is completely an intrinsic property of Sb^R^-LD which is evident from the fact that KD-Sb^R^-LD parasites behaves like their sensitive counterpart by virtue of losing its surface glycoconjugates with terminal sugar N-acetyl-D-galactosaminyl residue. But our observations do not exclude the possibility of other parasite derived factors that may contribute to differential immune modulation capacity of Sb^R^-LD parasites. Further characterization of the nature of the glycoconjugate is needed to understand the specific interaction with host immune system components.

There are other studies that establishes role of regulatory DCs in the induction of IL-10 from Th1 cells [58], [59], but the mechanism behind potent iTreg induction by Sb^R^-LD, remains under further investigation. This study allowed us for controlled experimentation and exploration of specific immune responses elicited by Sb^R^-LD infection which is difficult to achieve in actual human VL patients. The early infection process involves interaction of human immune system, the parasites themselves, promastigote secretory gel (PSG) [60] and the complex sand fly saliva with more than 50 active peptides and other components [61], [62]. This presents an enormous challenge for the researchers to dissect the role of the isolated individual players in this process and even more so, how these individual components interact with each other during natural infection process. Our *in vitro* system cannot address this bewildering complexity of natural infection process and may not exactly mimic the *in vivo* scenario. Studies with natural sand fly mediated infection cannot be extended to humans and we need to keep in mind the doubtful predictive value of animal models. In this context our work focuses on one isolated part of the early infection process when low inoculum of parasites interact with PBMCs with the emphasis on the differences in the intrinsic immune-modulation properties of pentavalent antimonial (Sb) sensitive and resistant *L. donovani* isolates. This may be one piece of the puzzle when it comes to the biology of Sb sensitive and resistant parasites and surely there are other components that are involved in the early infection process during natural transmission. Despite limitations of this model, it demonstrates how only a small number of parasites have potent immune-modulatory effect and can influence the local cytokine environment to suppress anti-parasitic activity; with drug resistant parasites having greater immune-modulation capacity. This knowledge about the immune-suppressive mechanisms associated with LD infection may allow us to design a more potent therapeutic approach to treat this dreaded disease.

## Supporting Information

Figure S1 **Similar parasite burden pattern in Sb^S^ and Sb^R^-LD-PBMCs.** The amount of percent infected adherent cells and number of amastigotes per hundred adherent cells were almost similar in both Sb^S^ and Sb^R^-LD-PBMCs (A,B,C and D). Freshly isolated PBMCs were incubated with Sb^S^ and Sb^R^-LD isolates on coverglass and after day two and day eight, coverglasses were washed, fixed with methanol and stained with Giemsa. The numbers of parasites in the adherent cells were enumerated after observing under light microscope. Data were analysed by Mann-Whitney test, and levels of significance are indicated by P values.
(TIF)

Figure S2 **Both Sb^S^ and Sb^R^-LD infection results in enhanced IL-27 production.** Level of IL-27 measured at early and late time point of interaction. Freshly isolated PBMCs were incubated with Sb^S^ and Sb^R^-LD isolates and after day two and day eight, culture supernatants (Sb^S^ and Sb^R^-sup) were collected and level of IL-27 was measured by ELISA. Median values were indicated (n = 10). Data were analysed by the Mann-Whitney test, and levels of significance are indicated by P values.
(TIF)
