# Supplementary figures and images for: Antimony Resistant Leishmania donovani but Not Sensitive Ones Drives Greater Frequency of Potent T-Regulatory Cells upon Interaction with Human PBMCs: Role of IL-10 and TGF-β in Early Immune Response
Source: PLoS Negl Trop Dis. 2014 Jul 17;8(7):e2995. doi: 10.1371/journal.pntd.0002995 (PMC4102415; doi:10.1371/journal.pntd.0002995)

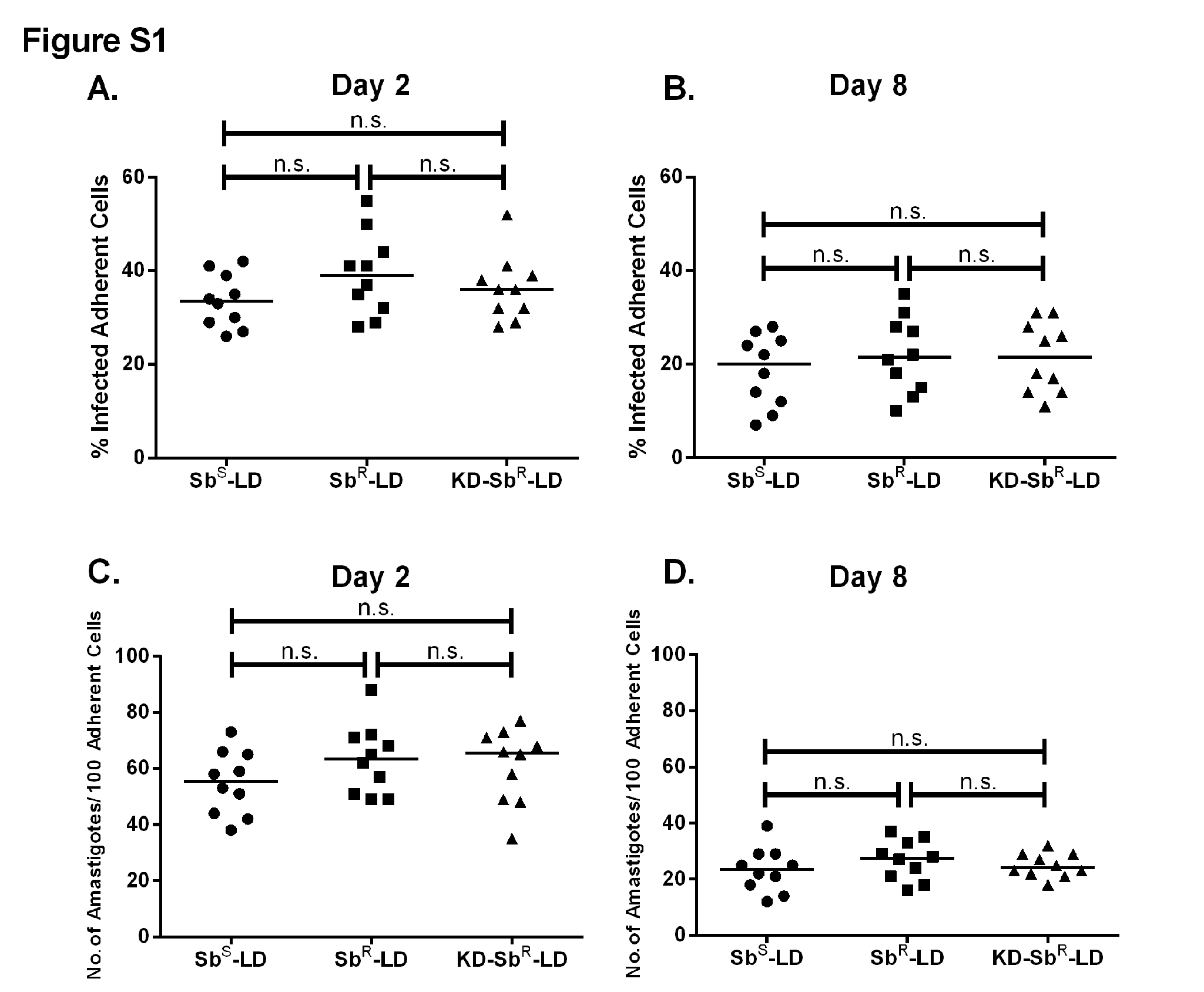

Supplement: Figure S1 — Similar parasite burden pattern in SbS and SbR-LD-PBMCs. The amount of percent infected adherent cells and number of amastigotes per hundred adherent cells were almost similar in both SbS and SbR-LD-PBMCs (A,B,C and D). Freshly isolated PBMCs were incubated with SbS and SbR-LD isolates on coverglass and after day two and day eight, coverglasses were washed, fixed with methanol and stained with Giemsa. The numbers of parasites in the adherent cells were enumerated after observing under light microscope. Data were analysed by Mann-Whitney test, and levels of significance are indicated by P values. (TIF) [file pntd.0002995.s001.tif]

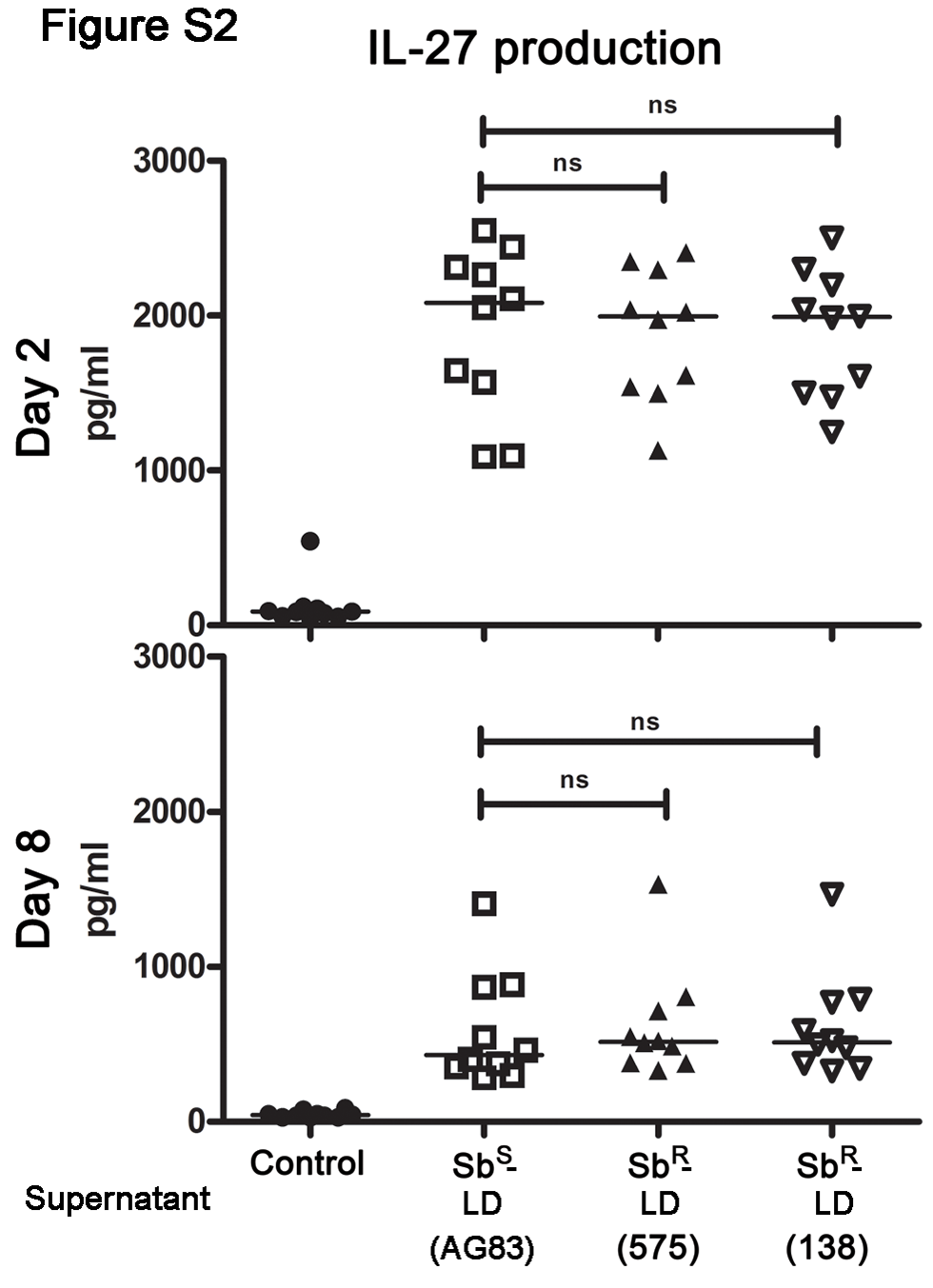

Supplement: Figure S2 — Both SbS and SbR-LD infection results in enhanced IL-27 production. Level of IL-27 measured at early and late time point of interaction. Freshly isolated PBMCs were incubated with SbS and SbR-LD isolates and after day two and day eight, culture supernatants (SbS and SbR-sup) were collected and level of IL-27 was measured by ELISA. Median values were indicated (n = 10). Data were analysed by the Mann-Whitney test, and levels of significance are indicated by P values. (TIF) [file pntd.0002995.s002.tif]
